# Longitudinal cohort study on subsequent injury risk in professional football players in the Qatar Stars League: a probabilistic approach using basic learning

**DOI:** 10.5114/biolsport.2026.152345

**Published:** 2025-10-07

**Authors:** Montassar Tabben, Karim Chamari, Khalid Alkhelaifi, Tanvir Alam, Jassim Almulla

**Affiliations:** 1 Aspetar Orthopedic and Sports Medicine Hospital, Doha, Qatar; 2 Olympic Medical and Scientific Committee, Qatar Olympic Committee, Doha, Qatar; 3 Naufar, Wellness and Recovery Center, Doha, Qatar; 4 Higher Institute of Sport and Physical Education of Ksar Said, University of Manouba, Manouba, Tunisia; 5 College of Science and Engineering, Hamad Bin Khalifa University, Doha, Qatar; 6 Qatar Football Association, Doha, Qata

**Keywords:** Reinjury, Subsequent injury, Athletic injury, Markov chains, Soccer, Prevention, Artificial intelligence, Machine learning

## Abstract

Better understanding of the biomechanical and physiological mechanisms underlying subsequent injuries could have substantial implications for clinical practice in sports medicine. We investigated subsequent injury risk among professional football players in the Qatar Stars League (QSL), focusing on injury recurrence patterns over nine competitive seasons (2013–2021). Through an observational cohort study, we collected data on time-loss injuries from 1,258 players, recording 4,700 injuries categorized by body part, injury type, and recurrence. Utilizing Markov model, we explored probabilistic links between initial/index and subsequent injuries (defined as those occurring within the same season), highlighting patterns of recurrence in muscle groups prone to biomechanical strain. Our analysis identified 1,599 injuries (34% of total) as subsequent, primarily affecting the thigh (notably hamstrings) and groin. For instance, hamstring injuries exhibited an 7.5% (± 1.3%) probability of recurrence within the same season, while groin injuries had a 2.9% (± 0.82%) probability of resulting in subsequent hamstring injury. Our findings suggest that even basic probabilistic modeling, such as Markov chains, can enhance targeted injury prevention strategies. The high rate of recurrence, particularly in lower limb muscles, underscores the need for tailored rehabilitation programs emphasizing biomechanical stability. This comprehensive study offers a robust evidence base for injury mitigation strategies in elite football, recommending proactive monitoring and data-driven interventions to reduce injury recurrence and enhance player health, availability, and long-term performance.

## INTRODUCTION

In sports medicine, previous injury has long been recognized as a critical risk factor for future injuries [1, 2]. Studies consistently show that athletes with a history of injury are more prone to experience further physical setbacks. However, a vital distinction must be made between two related but different concepts: “re-injury” and “subsequent injury.” Re-injury refers specifically to the recurrence of the same injury at the same anatomical location, while subsequent injury is any new injury, irrespective of its relationship to the original one; both within 12 months [3]. This classification is important due to the substantial prevalence of repeat injuries in sport. For instance, Bahr and Holme [4] reported that re-injuries can comprise up to

30% of all injuries in certain athletic populations, while Finch and Cook [3] noted a 13.7% rate of re-injuries within a season in elite Australian football.

Despite the growing body of literature in sports injuries, the challenge of predicting subsequent injuries remains under-explored. While re-injuries have been more frequently studied due to their direct connection to an initial trauma, subsequent injuries may be influenced by a wider array of factors, such as biomechanical imbalances or incomplete rehabilitation [4]. This gap in the literature leaves sports medicine practitioners with limited tools to accurately assess and mitigate risks of new injuries, related or unrelated to the index injury. Finch and Cook [3] stated that a better understanding of nature, causes and outcomes of subsequent injuries would potentially have substantial implications for clinical practice in sports medicine where preventing future injuries and recovery to preinjury function are the major goals of treatment.

Machine Learning (ML) has emerged as a promising solution to address complex challenges. By leveraging vast datasets and identifying hidden patterns, ML techniques are capable of advancing our understanding of injury risk profiles [5–7]. Several recent studies have explored the use of ML algorithms to assess injury risk with encouraging results. For example, Rommers et al. [8] applied XGBoost in elite youth players and reported 85% prediction accuracy, while Oliver et al. [9] used decision trees to highlight biomechanical markers of injury risk with an AUC of 0.66. While these studies demonstrate the potential of data-driven modeling for injury profiling, they differ substantially from our study in terms of population, methodology, and objectives. In contrast, our study employs a probabilistic modeling approach to examine subsequent injury risk occurring in the same season as index injury. For this, we have prospectively followed in professional football players over nine competitive seasons in the Qatar Stars League (QSL). This approach enabled us to explore the temporal patterns and transitions between initial and subsequent injuries within a single season, offering practical insights for injury prevention and rehabilitation strategies [9].

## MATERIALS AND METHODS

### Study design

This is a longitudinal cohort study collecting injury data using the Sport Medicine Diagnostic Coding System (SMDCS) to classify injuries [10]. Coding was performed by the team physicians of the teams using standardized definitions and a study manual [11].

### Setting and participants

We prospectively recorded individual time-loss injuries in adult (≥ 18 years old) male professional footballers from Qatar through 8 seasons (July 2013 through May 2021, included). Seventeen teams (12 first and 5 second division league) were followed throughout the domestic season, as well as periods of international camps or tournaments. We included teams that provided at least six consecutive months of data and fulfilled the minimum standard for data quality [11]. We included players being either a first-team squad member or training regularly with the first team. Players with preexisting injuries at the start of each season were included in the study only after successful return to play from these conditions. Players newly recruited to a club were included from their recruitment date.

Players who did not meet these inclusion criteria (e.g., limited availability, not affiliated with the first team, or incomplete data reporting) were not enrolled in the injury surveillance protocol and therefore not recorded in the dataset [12, 13]. The team physician in each club oversaw data collection using standardized tools [11, 12]. A detailed study manual was provided to each club’s medical staff at enrollment, outlining injury definitions, coding procedures, and data entry protocols. We recorded data using a custom-made Microsoft Office Excel^®^ file for quick data entry. Injury cards were also provided in Microsoft Office Word^®^ to assist clinicians in taking notes during daily clinical activity, prior to entry into the master data file. In addition, demonstration sessions were conducted whenever a new team physician joined the program to ensure standardization in data collection. These efforts were intended to maintain consistency and reliability in reporting across all participating teams throughout the study period. We asked the clubs to submit their data every month by email. According to the IRB approved research protocol (ADLQ-IRB: E2017000252), team physicians or physiotherapists verbally informed all players about the purposes and procedures of the study and the latter provided verbal consent before being included in the study [11].

### Data sources

To facilitate comparison with previous studies, injury definitions and data collection procedures followed the 2006 consensus statement on epidemiological studies in football and the IOC model for recording illnesses [13, 14]. The results are reported according to the 2020 IOC consensus statement on injury and illness epidemiology [14].

We recorded all injuries resulting in a player being unable to fully participate in training or match play (i.e., time-loss injuries). The player was considered injured until the team medical staff allowed full participation in training and availability for match selection. We did not record injuries that did not cause time off from football activities, nor injuries occurring outside football activities.

### Variables used in the study

We recorded the following characteristics for each injury: Diagnosis, onset (sudden vs. gradual), severity (number of days of time loss), injury: (i) type, and (ii) involved body part, (iv) index injury (first injury of any type recorded during the study), exacerbation (re-injury happening before the return to play), or re-injury (injury to the same body part and same structure type occurring after return to play, and within one year from index injury), (v) training or match injury (cases of gradual-onset injuries, where the injury could not be clearly attributed to a specific session, were classified as not applicable) (vi) whether the injury is caused by contact or not. We have also classified the injuries based on a specific diagnosis using common diagnosis in football [15]. Subsequent injuries were defined as any new time-loss injury occurring to the same player within the same competitive season as the index injury. Seasons were treated independently to avoid confounding effects of the summer break.

### Data Pre-processing

Before analyzing the data, the data had to be pre-processed using different methods prior to working on the dataset using Python programming. In our study, we cleaned all the injuries naming to use consistent naming across the dataset. We also removed records of players’ injuries with only one injury because it indicates that there was no previous injury for the player within the same season. We also excluded records where the injury was caused by contact. We also looked for records with missing data and removed any record that has missing “Player ID” or “Body Part” as they are crucial to identify if there has been a previous injury or not.

Finally, we analyzed the data to decide if there was any data that could be combined and we combined the data of the “body part” with “body part side” into the body part field; in this case, the same body part with different body side would be considered as another body part in our analysis (e.g. left thigh and right thigh would be considered different body parts).

### Data Analysis

After completing the data pre-processing step, we proceeded to the data analysis phase. We began by calculating the overall probability of each injury type by dividing the number of occurrences of that injury by the total number of recorded injuries. Next, we computed the conditional probability of a subsequent injury given a previous injury, using a first-order Markov model to estimate the likelihood of transition from one injury type to another within the same season [16].

We applied a first-order Markov model [16] to analyze the probability of transitions between injury states within the same season. Transition probabilities were calculated based on the observed frequency of injury sequences for each player. Specifically, we defined a “state” as a combination of the body part and injury nature, and a transition occurred when a subsequent injury was recorded after an initial injury within the same season. Transition probabilities were estimated by dividing the number of observed transitions from one state to another by the total number of transitions from the initial state. We analyzed only the first subsequent injury for each initial injury to ensure a clear sequence without confounding effects. To ensure focus on clinically meaningful patterns, we excluded transitions with probabilities below 0.002. Confidence intervals for each transition probability were computed using binomial proportion methods. This approach enabled us to identify statistically and clinically relevant subsequent injury patterns with quantified uncertainty.

Following this, we grouped the data into three major scenarios of subsequent injuries as proposed in Finch et al. [3] and Toohey et al. [17]:

1. **Injury in the Same Body Part and Same Nature:** This scenario covers injuries, where a player had *a* subsequent injury occurred in the same body part but both injuries were of same in nature (this is the specific case of re-injuries).
2. **Injury in the Same Body Part with Different Nature:** This scenario covers injuries, where a player subsequent injury in the same body part and both injuries were different in nature.
3. **Injury in any Categorization:** this scenario covers injuries where a player had subsequent injury that could be of any (same or different) categorization.

This model enabled us to estimate the likelihood of transitioning from one injury type to another, according to the specified scenarios. The approach provided valuable insights into significant patterns in injury progression.

Confidence intervals were calculated to reflect variability within the sample, with a transition probability of 0.10 having a 95% confidence interval of approximately (0.098, 0.102).

### Ethical Approval for the Study

The study has been approved by the Institutional Ethical Committee with reference number ACS/0000207/ak.

## RESULTS

**FIG. 1:**
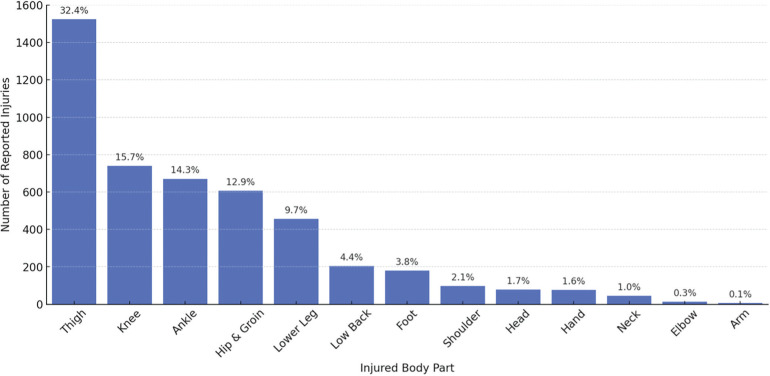
Distribution of Injuries by Body Part (n=4700 injuries)

Over the nine seasons studied, there were a total of 4,700 injuries recorded for 1,250 players. Injuries by body parts are presented in Figure 1, which shows the distribution of the most commonly injured body parts: thigh (32.4%), knee (15.7%), ankle (14.3%), hip and groin (12.9%), and lower leg (9.7%).

From these 4,700 injuries, 1,599 were identified as subsequent injuries. Overall, 268 players sustained at least one subsequent injury during the study period, with an average of 1.7 (± 1.1) subsequent injuries per player per season. These subsequent injuries were analyzed according to various scenarios described in the Methods section, using the first-order Markov model to compute the transition probabilities between injury types.

**FIG. 2:**
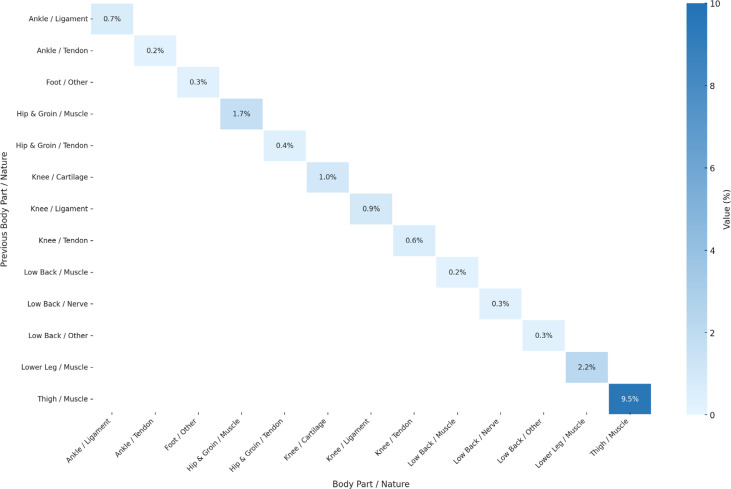
Probability distribution of subsequent injury for same body part and same nature considering previous n (=1) injury, showing relation between body parts and nature. Darker blue colors indicate greater influence (probability) of previous injury on the subsequent injury. The graph excludes injuries below 0.2% probability from the result. Sample Size: 1,599 cases of subsequent injuries

**FIG. 3:**
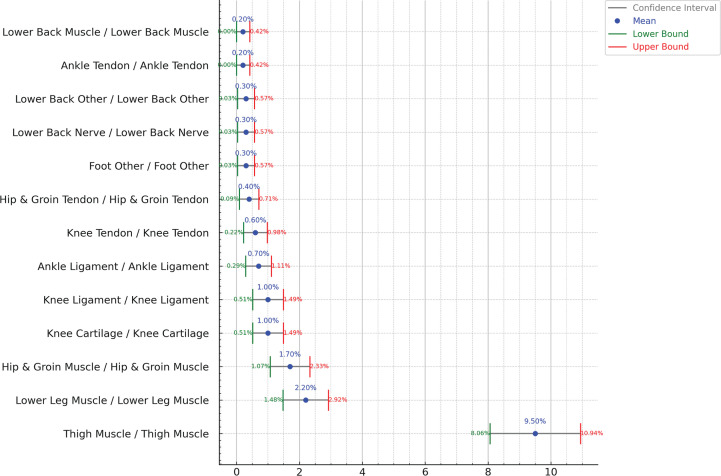
Confidence Intervals for subsequent injuries with same body part and same nature considering previous n (=1) injury, showing relation between body parts and nature. Red color text shows low intervals, green text is the high interval and the blue is the median.

Figure 2 shows injuries occurring in the same body part with the same nature (re-injuries). The analysis revealed that re-injuries are more frequent than general subsequent injuries and are more frequent in the Thigh Muscles (9.5%) with confidence interval of ± 1.4%. For Lower Leg Muscles (2.2%) with confidence interval of ± 0.7%. The remaining subsequent injuries have probability below 2% as shown in Figure 2. Figure 3 shows the confidence interval for all reinjuries.

**FIG. 4:**
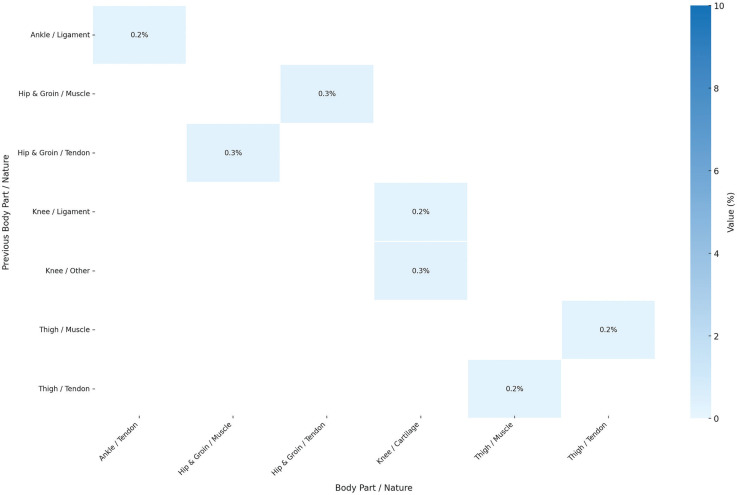
Probability distribution of subsequent injury for same body part and different nature considering previous n (=1) injury, showing relation between body parts and nature. Darker blue colors indicates greater influence (probability) of previous injury on the subsequent injury. The graph excluded injuries below 0.2% probability from the result. Sample Size: 1,599 cases of subsequent injuries

**FIG. 5:**
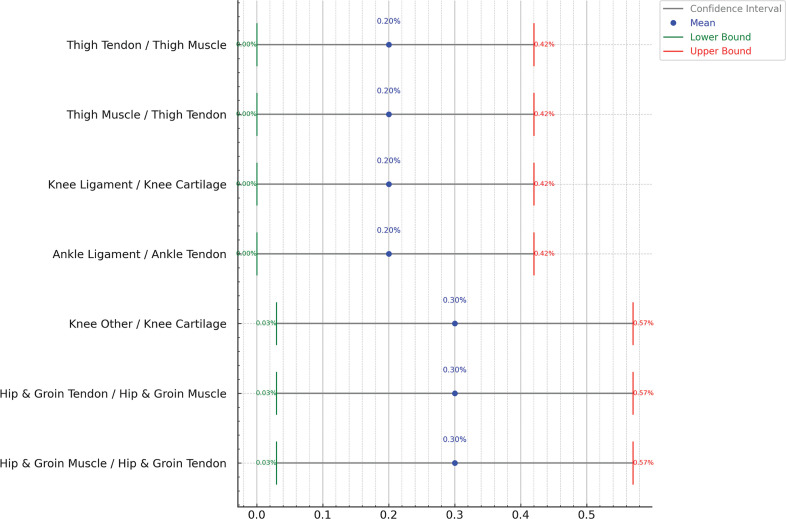
Confidence Intervals for subsequent injuries with same body part and different nature considering previous n (=1) injury, showing relation between body parts and nature. Red color text shows low intervals, green text is the high interval, and the blue is the median.

Figure 4 explores injuries that occurred in the same body part but with a different nature from the previous injury. The findings show that these injury combinations are quite rare such as Hip and Groin Tendon Injury occurring subsequently to Hip and Groin Muscle Injury (0.3%) with confidence interval of ± 0.3%. Other injury combinations are shown in Figure 4. Figure 5 shows all the confidence interval for the subsequent injuries to the same body part but different nature.

**FIG. 6:**
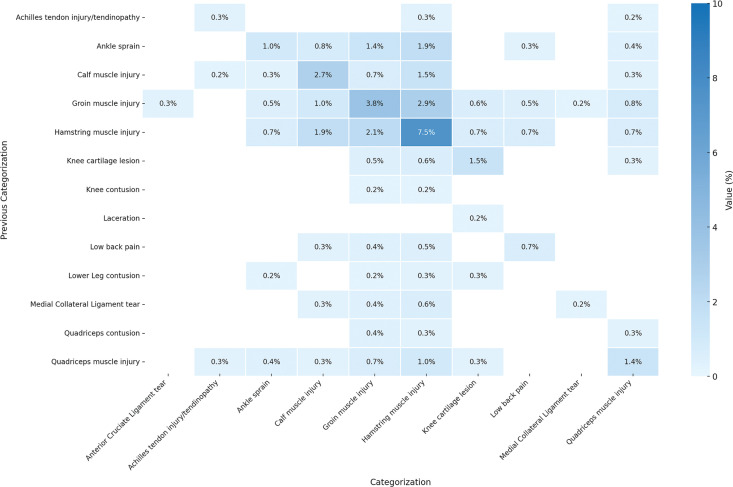
Probability distribution of subsequent injury for same or different categorization considering previous n (=1) injury. Darker blue colors indicates greater influence (probability) of previous injury on the subsequent injury. The graph excludes injuries below 0.2% probability and injuries with “Unsure” and “Others” categorization from the result. Sample Size: 1,599 cases of subsequent injurie. MCL: Medial Collateral Ligament. ACL: Anterior Cruciate Ligament.

**FIG. 7:**
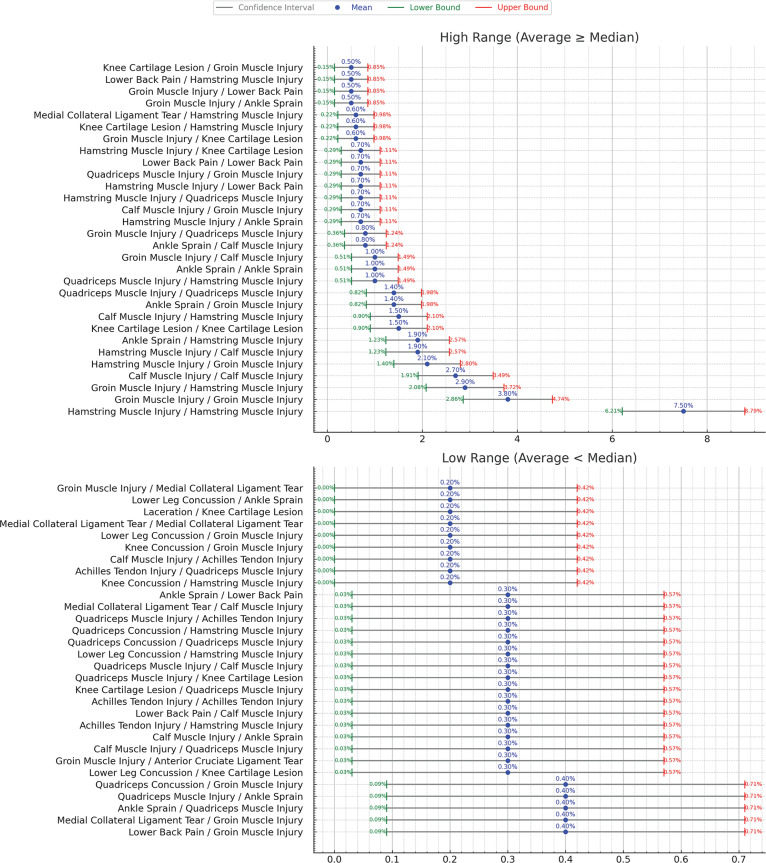
Confidence Intervals for subsequent injuries with same or different categorization considering previous n (=1) injury. Red color text shows low intervals, green text is the high interval and the blue is the median.

Figure 6 expands the scope to include injuries with same or different categorization. The most common categorization is injuries categorized as “Hamstring Muscle Injury” with a previous injury of the same categorization which has 7.5% probability and ± 1.3% confidence interval. Figure 7 shows the confidence interval for the cases for same or different categorization.

## DISCUSSION

We analyzed subsequent injuries in elite footballers to cover injuries that have at least one subsequent injury that occurred during the same season, excluding contact mechanism’ injuries. We report that 34% of all the injuries from the nine analyzed seasons followed such a subsequent injury logic (1,599 out of 4,700). Subsequent logic does not automatically imply any causality link, however, our analysis showed patterns of subsequent injuries in relation to previous same season’ injuries. Indeed, an injury to the thigh muscle had 9.5% (± 1.44%) likelihood of resulting in a subsequent injury of any nature to the same thigh muscle (Figure 2). When considering a more specific diagnosis, the most frequent injury in football, i.e. hamstrings muscle injury, showed a pattern of subsequent injury (re-injury) with a probability of 7.5% (± 1.29%) happening in the same season (Figure 6). The latter same re-injury pattern also applied to another frequent injury in football, i.e. groin injury, with a probability of 3.8% (± 0.94%) (Figure 6). Another subsequent injury pattern concerns hamstring muscle injuries that occurred subsequently to a groin injury with 2.9% (± 0.82) probability.

There is a growing interest in applying machine learning (ML) techniques to predict and prevent sports injuries, particularly in highperformance settings. Prior studies have explored deep learning and computer vision methods in contexts such as gymnastics, basketball, and winter sports [18–20]. While these models demonstrate high classification accuracy, they often require large and complex datasets and may lack interpretability for clinical application. In contrast, our study contributes to this data-driven movement by employing a simpler probabilistic approach—Markov chains—to model subsequent injury risk. Though not an advanced ML method, Markov modelling offers transparency and clinical interpretability, making it a pragmatic tool for identifying meaningful injury patterns within elite team sports settings. A similar profiling approach has been applied in a recent study on injury patterns among male police cadets, providing useful comparative insight into structured physical training environments [2].

The finding that 34% of the injuries followed a subsequent injury pattern is relatively high but not surprising. Indeed, Finch and Cook [3] reported as much as 13.7% of re-injuries in the same season in Australian football. As our results show, the subsequent injuries pattern we observed is very close to the concept of ‘’re-injury’’. Indeed, most of the salient likelihood (above 10%) of our results concern subsequent injuries occurring to the same body part with same injury nature. From a clinical perspective, these findings highlight the critical importance of targeted rehabilitation programs that extend beyond standard return-to-play protocols. Given that subsequent injuries predominantly occur at the same site and with the same nature, rehabilitation strategies should emphasize long-term tissue resilience rather than merely restoring pre-injury function; ideally following a joint-by-joint approach as recently advocated by Dhahbi et al. [21]. In addition, strength and neuromuscular control exercises tailored to the injured area should be progressively integrated into both rehabilitation and post-rehabilitation phases to mitigate the risk of re-injury.

Furthermore, the low likelihood of injuries to antagonist muscles (e.g., an index quadriceps injury followed by a hamstring injury with a 1.0% likelihood) suggests that neuromuscular imbalances may not be the primary driver of subsequent injuries in elite football. However, these occurrences still warrant biomechanical assessments to identify compensatory movement patterns that could predispose athletes to additional injury risks. This aligns with recent studies advocating continuous biomechanical monitoring and movement retraining as preventive measures in elite sports [20, 22]. The latter might be explained by a biomechanical pattern change. We showed that the pattern involving different body parts was less likely to occur. For instance, a groin muscle injury had 2.9% (± 0.82%) likelihood of being followed by a hamstring muscle injury, while the likelihood of an inverted order of these injuries was of 2.1% (± 0.7%). There seems to be a chain’ effect of relationship between different body parts that are in proximity [22]. A hamstring muscle injury was followed by a calf muscle injury with a 1.9% (± 0.67%) likelihood. However, proximity is not always present in many other cases. For instance, an ankle sprain was followed by a hamstring muscle injury with a likelihood of 1.9% (± 0.67%) as well. In the latter case, there is a possibility that the ankle sprain would have resulted in a biomechanical pattern change, overloading the hamstrings and increasing their likelihood of being injured. This relationship could be explained by the concept of arthrogenic muscle inhibition (AMI), a neural response that alters muscle activation following joint injury. Research suggests that AMI can lead to neuromuscular dysfunction, affecting proximal muscle groups and increasing injury susceptibility. These findings align with the broader call for injury prevention programs that emphasize biomechanical resilience and neuromuscular reconditioning [23].

For example, Sedory et al. [24] demonstrated that individuals with unilateral chronic ankle instability exhibit bilateral arthrogenic inhibition of the hamstring muscles, leading to reduced activation and impaired neuromuscular control. This inhibition may contribute to altered lower-limb biomechanics, resulting in increased strain on the hamstrings during dynamic movements such as sprinting or sudden changes in direction. The reduced neuromuscular efficiency in the hamstrings could predispose them to subsequent injury, particularly in high-intensity activities common in football. In this regard, Xu et al. [22] reported that participants with Chronic Ankle Instability had altered lower extremity proximal joint movement strategies during side cut, stop jump, and landings, suggesting that such alterations probably increase the risk of ACL (anterior cruciate ligament) injury. This has been confirmed by He et al. [25] in their systematic review, showing that during landing tasks, patients with chronic ankle instability had (i) increased hip extension- and knee extension-moments, (ii) decreased hip flexion angles, (iii) increased peak vertical ground reaction forces, and (iv) increased trunk lateral flexion angles. All latter biomechanical variables are associated with higher risks of enduring an ACL injury. Such ‘’distal’’ relationships in subsequent injury patterns have been established with injuries subsequent to concussions. Indeed, Chou et al. [26] showed that following a recent concussion (within 2 months), individuals displayed a slower locomotion-related performance and decreased postural stability, which are associated with a higher risk of lateral ankle sprain. Moreover, within 2-year post-concussion, after jumping, individuals adopt a more erect landing posture coupled with higher knee internal adduction moment, both of which are risk factors for ACL injury.

### Study limitations and Future Work

Our choice to limit subsequent injuries to the same season may have introduced selection bias, as injuries occurring late in a season could still influence injuries in the following season. This cutoff might underestimate true recurrence rates and obscure longer-term injury patterns. Future studies should consider tracking injuries across multiple seasons to capture a more comprehensive view of subsequent injury risk. Such cases were numerous in our database, but we did choose to exclude them from our study. Indeed, we considered that the offseason would impact any link between injuries. However, as several elite football teams are involved in continental competitions, and some elite players are also additionally solicited by their national teams, there is often no off-season for many players. In such cases, we believe that the analysis of subsequent injury shall not be interrupted from season to season. Moreover, our study is limited to only one previous injury as a cause for a subsequent injury. Future studies might expand the analysis to eventually include more than one previous injury using the Markov Model. Another limitation is the small data sample used in the study which is not powerful enough to conduct a deep learningbased study. We therefore call for multiple research centers to join their efforts in producing larger data sets, based on consistent definitions and data collection methods [14]. Moreover, Markov models are typically used to model the likelihood of transitions between states, but they have some limitations. Markov models struggle with timedependent transitions, making it difficult to account for injuries that worsen or recover at varying rates depending on the length of time spent in a particular state. For example, recovery time from an injury may increase the probability of re-injury, but a simple Markov model cannot capture these dynamics effectively. Another key issue is the state space explosion, where the number of possible states increases dramatically as the system becomes more complex. This is particularly problematic in injury analysis, where many variables like injury type, severity, and location shall be considered. However, since this paper focuses on just one previous injury, the complexity of the data and states is not large, making the Markov model a suitable choice for this specific application. Future alternatives like semi-Markov models address time-dependent transitions, and Bayesian models offer more dynamic predictions when dealing with larger, more complex data sets.

In addition, our study may be subject to several uncontrolled confounding factors. These include variations in player workload and match exposure, which were not uniformly recorded across clubs, and may influence both injury occurrence and recurrence. Differences in rehabilitation protocols, training load management, and medical practices across teams could also have influenced injury outcomes. Environmental factors such as pitch surface quality or travel-related fatigue may contribute further to the complexity. Additionally, unmeasured psychological variables, including motivation, stress, or pressure to return, may affect injury recurrence independently of biomechanical or physiological readiness. These factors should be considered when interpreting the probabilistic patterns observed, and future studies with more detailed covariate tracking are warranted.

## CONCLUSIONS

We conclude that our study provides robust, longitudinal evidence on subsequent injury patterns that can inform clinical decision-making and prevention strategies in elite football. Indeed, prevention and rehabilitation programs should consider the substantial likelihood of subsequent injury that we have showed for thigh muscles, with specific focus on hamstrings and groin muscles as the index injury that might result in subsequent injury to the groin or hamstrings muscles.
